# Improving preparation in the emergency trauma room: the development and impact of real-time data transfer and dashboard visualization system

**DOI:** 10.1007/s11548-024-03256-2

**Published:** 2024-09-11

**Authors:** Anna Schatz, Georg Osterhoff, Christoph Georgi, Fabian Joeres, Thomas Neumuth, Max Rockstroh

**Affiliations:** 1 Innovation Center Computer Assisted Surgery, Faculty of Medicine, University Leipzig, Leipzig, Germany; 2 Department of Orthopaedics, Trauma and Plastic Surgery, Leipzig University Hospital, Leipzig, Germany; 3 Department of Simulation and Graphics, Otto-Von-Guericke-University Magdeburg, Magdeburg, Germany

**Keywords:** Emergency trauma room, Pre-notification, Pre-hospital information, Visualization system, Real-time data transfer

## Abstract

**Purpose:**

This study examines, with clinical end users, the features of a visualization system in transmitting real-time patient data from the ambulance to the emergency trauma room (ETR) to determine if the real-time data provides the basis for more informed and timely interventions in the ETR before and after patient arrival.

**Methods:**

We conducted a qualitative in-depth interview study with 32 physicians in six German and Swiss hospitals. A visualization system was developed as prototype to display the transfer of patient data, and it serves as a basis for evaluation by the participating physicians.

**Results:**

The prototype demonstrated the potential benefits of improving workflow within the ETR by providing critical patient information in real-time. Physicians highlighted the importance of features such as the ABCDE scheme and vital signs that directly impact patient care. Configurable and mobile versions of the prototype were suggested to meet the specific needs of each clinic or specialist, allowing for the transfer of only essential information.

**Conclusion:**

The results highlight on the one hand the potential need for adaptable interfaces in medical communication technologies that balance efficiency with minimizing additional workload for emergency medical services and show that the use of pre-notification systems in communication between ambulance and hospital can be supportive. Further research is recommended to assess practical application and support in clinical practice, including a re-evaluation of the enhanced prototype by professionals.

## Introduction

In Germany, the number of ambulance rescues is increasing. Due to the severity of the injuries or the impairment of vital parameters, patients must be transferred from the scene of accident to the hospital. The efficient transfer and use of medical and patient data is crucial for a fast and precise further treatment of the patients [1, 2]. Upon arrival at the hospital, patients with severe injuries are directed to the emergency trauma room (ETR) of trauma centers. These centers are organized into three levels based on their capabilities, expertise, and infrastructure: local (LTC), regional (RTC), and supra-regional (STC). LTCs provide initial comprehensive care and can transfer patients to higher-level centers if necessary. RTCs and STCs provide extensive emergency services and definitive care. STCs, situated in maximum care hospitals, are equipped to handle more complex cases due to their larger capacities and advanced facilities. This tiered system optimizes the allocation of medical resources and ensures that patients receive the most appropriate care, according to the severity of their injuries. It facilitates immediate treatment at LTCs and enables more complicated cases to be escalated to RTCs or STCs [3, 4]. For immediate medical intervention in the ETR of a trauma center, effective communication within the team and with the emergency medical services (EMS) is essential. A pre-notification by EMS of the patient’s condition can facilitate patient preparation by activating and informing ETR staff prior to the patient’s arrival. The ETR team is thus able to prepare clinical procedures in advance, such as mobilizing certain team members and preparing for necessary medical interventions. These measures can reduce treatment delays and improve the efficiency of the process for both the patient and the ETR team [5, 6].

A significant challenge between EMS and hospitals is the frequent and often inadequate communication between the two entities. The current practice of EMS providing advance notice by telephone is susceptible to error. It is challenging to communicate dynamic changes in the patient’s condition or arrival time effectively, as each change necessitates another call. Furthermore, the presence of loud background noise during the call can impede the transmission and comprehension of information. The loss of information can result in the interruption of clinical processes, which may lead to delays in treatment and a reduction in the quality of patient care [6–8]. System such as NIDAklinik, Corpuls.mission and Mobimed Unity focus on improving data transfer between emergency services and hospitals. Increasing demands for efficient and effective patient care require an advanced system that helps transmit patient data. Corpuls.mission (GS Elektromedizinische Geräte G. Stemple GmbH, Kaufering, Germany) enables real-time transmission of medical, audio and video data directly from the scene of an accident to tele-emergency physicians, supporting telemedicine consultations in emergency situations. In this system, the corpuls C^3^ (defibrillator/monitor with telemetry module) transmits vital signs and electrocardiogram data (ECG) [9]. The NIDA system (ZTM, Bad Kissingen, Germany) which was developed from the Stroke Angel program, offers telemedical pre-notification by EMS. This system transmits vital signs, scene images, and patient data to prepare emergency rooms for incoming cases, improving the readiness and response efficiency of medical teams [10]. Another solution is Mobimed Unity (Ortivus AB, Danderyd, Sweden). This intelligent ambulance service enables the exchange of real-time data, such as vital signs and ECG, to give the physicians in the hospital an overview of the patient's condition in the ambulance [11].

This paper is part of the completed research project MOMENTUM aimed at investigating the networking of medical devices and software components within an ambulance and between ambulance and hospital using the 5G mobile communication technology. The objective of this project was to ascertain whether 5G for internal networking provides advantages for EMS. The continuous availability of clinical data throughout the entire process, from the accident site to the ETR to the operating room, provides the opportunity to optimize patient care and enhance the efficiency of treatment processes. As part of this project, a flexible and secure communication architecture has been developed. The advantage of this communication architecture over the other systems mentioned is the open communication standard IEEE 11073 Service-oriented Device Connectivity (SDC) with its ability to integrate different medical devices from different manufacturers into a unified network that enables real-time transmission of patient data from the ambulance to the ETR [12, 13]. In 2022, a former ambulance has been modified with the addition of supplementary medical devices, which have been incorporated into an integrated communication infrastructure based on a 5G-Campus-Network, WiFi and Ethernet. In order to demonstrate the advantages of the 5G network structure, a visualization system was developed that displays the real-time patient data recorded in the ambulance on a large screen in the hospital's ETR.

The aim of this paper is to evaluate with clinical end users which features of live medical and patient data transmission are relevant for the emergency team and how they should be presented to enable a better emergency workflow. This will be done to determine if this visualization system will provide tangible benefits to ETR staff. Furthermore, the outcomes of this study will be utilized in the enhancement of the visualization system. In this paper, the visualization system is referred to as dashboard prototype. It is important to mention that for the visualization in the dashboard prototype, the origin of the data is irrelevant, whether it comes from the ambulance or from other clinical areas. Our state-of-the-art connectivity enables flexible and robust integration of this data. This ensures that ETR staff can access the information they need to optimize and support the emergency process. The implementation of the main technical infrastructure and the underlying requirements analysis are not part of this publication. Preliminary results from a subset of the sample have already been presented at a national conference focusing on what pre-hospital information is needed by physicians to ensure good resource preparation in the ETR for incoming trauma patients [14].

## Methods

In a qualitative in-depth interview study, physicians in the ETR were asked about specific features of a potential visualization system for pre-notification. The use of this dashboard prototype in the study allowed the physicians to provide their evaluation and feedback based on a practical and clear representation of the real-time patient data.

### Participants

Thirty-two (32) physicians that were part of the ETR team, from six hospitals in Germany and Switzerland were included in this study. The data collection took place from May 2022 to May 2023. The selected hospitals represent trauma centers from all three levels of care, including both urban and rural areas (Table 1).

**Table 1:** Physicians by professional title, department and working experience

Professional title	N	Department	N	Working experience	N
Chief physician	7	Trauma surgery	17	0–5 years	3
Senior physician	14	Anesthesiology	8	6–10 years	11
Medical specialist	4	Radiology	2	11–20 years	12
Assistant physician	7	General surgery	1	21–30 years	6
		Visceral surgery	1		
		Internal medicine	1		
		General medicine	1		
		Neurology	1		
	32		32		32

STC: Leipzig University Hospital (Germany)
  University Hospital Zurich (Switzerland)
RTC: Military Hospital Berlin (Germany)
  Sana Clinic in Borna (Germany)
LTC: Asklepios Clinic Uckermark in Schwedt/Oder (Germany)
  Evangelical Hospital Paul Gerhard Stift in Lutherstadt Wittenberg (Germany)

### Material

**Fig. 1:**
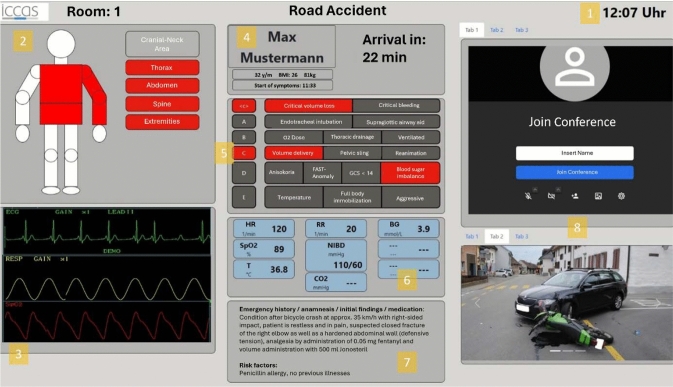
Dashboard prototype for visualization of real-time data transmitted from ambulance. Single features marked with numbers

The prototype, as one possible value-added service, integrates different medical devices into one comprehensive communication infrastructure. The initial prototype was developed based on previous individual interviews with physicians and observations from the hospitals (Fig. 1). To be able to evaluate this first prototype, the interviewed study was conducted.

**Fig. 2:**
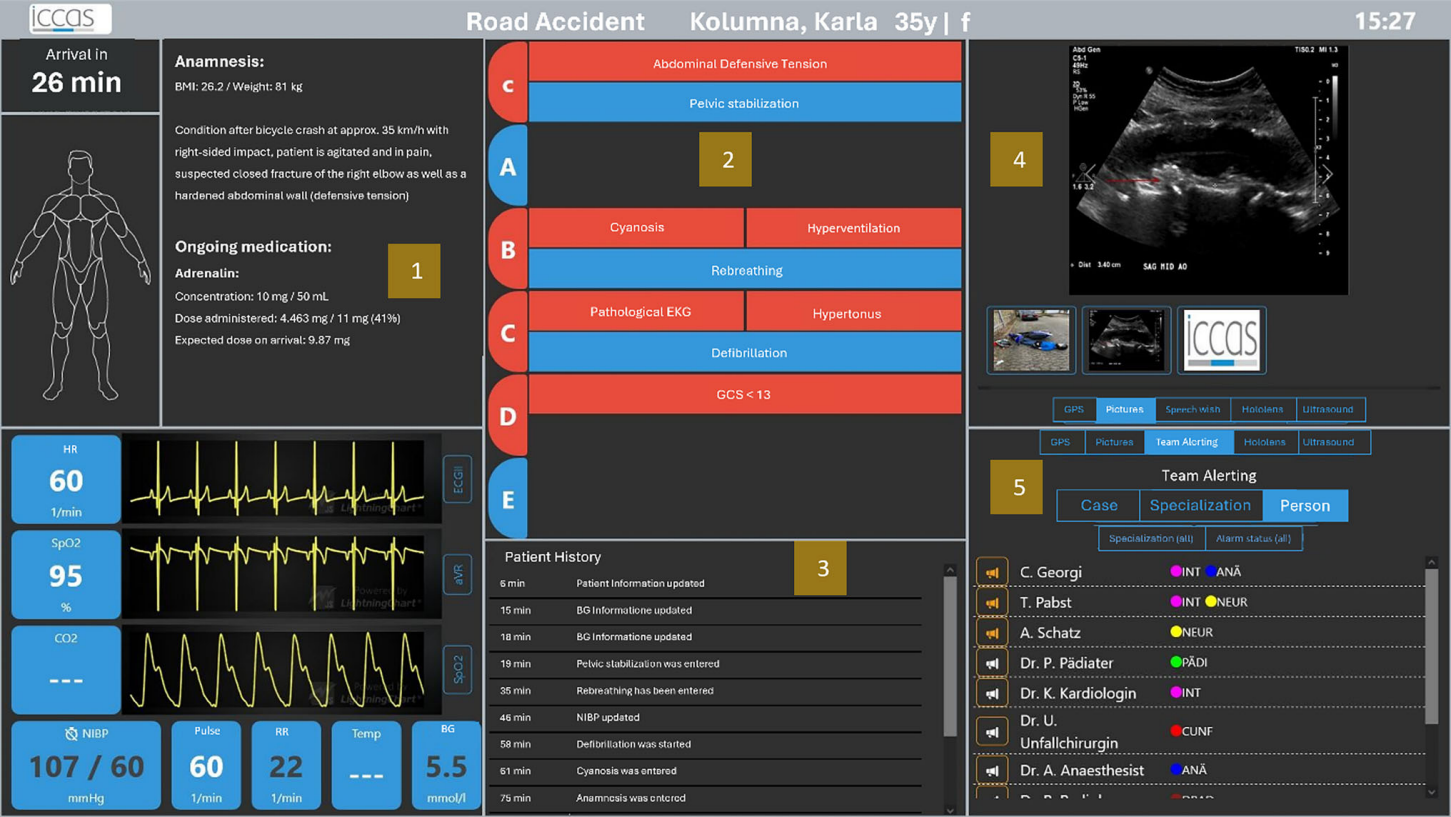
New dashboard prototype at the end of the project, considering the interview results

This prototype consists of three columns, each displaying specific information. The information is acquired from various data sources located in the ambulance. The top line (1) provides information on the transport diagnosis, current time and number of the ETR. The figure (2) illustrates the injured areas on the patient's body. The ECG (3) is displayed as a waveform. In addition, some vital parameters (6), e.g. heart rate, oxygen saturation, are displayed as numerical values. Both the ECG and the vital signs are transmitted by the patient monitor Infinity M540 (Drägerwerk AG & Co. KGaA, Lübeck, Germany). The patient information (4) includes general information such as name, age, and time of arrival. The ABCDE scheme (5) is a well-established algorithm for treatment in the ETR [15]. The emergency history (7) provides insight into allergies, pre-existing conditions and risk factors. This information is transmitted from the NIDApad (medDV GmbH, Fernwald, Germany), a tablet used by EMS for documentation purpose. At the time of device integration, live transmission of NIDApad data was not possible. The multimedia section (8) is divided into two parts, each featuring configurable tabs that display accident images, sonography images, and a video call option for communication between the ambulance and the hospital. The sonography images are captured using the ultrasound scanner C3 HD3 (Clarius, Vancouver, Canada), while the photos can be captured using the NIDApad. This dashboard prototype combines the connection of multiple data sources, e.g. NIDApad and patient monitor M540, and a consistent visualization of these data sources on one interface. The prototype was presented to participants using simulated data representing changes in patient information and displayed using a time loop mechanism. The red markers are meant to help and show what is currently relevant for certain values such as time of arrival, risk factors, ABCDE scheme, and vital signs. The prototype was showcased on a 13.3-inch screen to the test participants. The research method used was the semi structured interview. For this purpose, a guideline with questions for the interview was created. The interview questions were based on the dashboard prototype. The guideline of questions was tested and then improved and finalized. The interview questions are described in the following section.

### Procedure

The interviews were divided into two parts, evaluation of the features and evaluation of the visualization.

*Evaluation of the features.* At the beginning, each feature of the dashboard prototype was explained in detail. The participants were then asked questions to evaluate the features. In this first part of the interview, the participants had not yet seen the dashboard. The reason was to avoid pre-influencing, as the goal was to first determine the value of the features without the visualization. There were also questions about features that had not yet been implemented in the prototype. This feature set includes an *alert feature* to notify ETR staff, a *notification feature* for marking changes on the prototype, and a *patient history window* with time stamps displaying past changes of patient's health status such as vital sign fluctuations or new therapeutic steps, etc. (Table 2).

**Table 2:** Questions for evaluation of the features

Feature	Questions
Injured Figure, ECG, Patient Information, ABCDE Scheme, Vital Signs, Emergency history, multimedia section, alert feature, patient history window	Do you see any benefit in this? If yes/no why? Are there scenarios in which this information would help you? If yes, which ones and why? If no, why not?
Notification feature	Do you see any benefit in this? Why? How would you like to be notified? For which changes on the dashboard would you like to receive notifications?

*Evaluation of the visualization.* After assessing the functionalities, participants were asked to evaluate the visualization of the prototype. In the prototype exploration, they reported any thoughts that came to their mind when looking at the prototype. If the participants struggled to report what they saw, the interviewer encouraged them by asking questions. Furthermore, additional features that had not been implemented in the existing prototype were asked. This included the potential for a *mobile version* of the dashboard accessible via mobile devices, and the capability for personalizing the information displayed on the dashboard as *individual view* (Table 3). The interviews were conducted by the same person at the different hospitals. When time permitted, a second person was present to take notes. The interviews were recorded after the participants had signed the declaration of consent for participation and data protection.

**Table 3:** Questions for evaluation of the visualization

Feature	Question
Prototype exploration	What was the first thing you noticed? What are you looking at right now? etc
Mobile view	Would it be helpful for you to see information like this on a mobile device (smartphone, tablet) that you always carry with you? What information would that be?
Individual view	What would you think if each trauma leader on duty could customize the view of the dashboard, depending on which information is more important to him/her?
Last question	Do you have any other ideas or requests?

### Data analysis

The basis of the evaluation was the thematic analysis in order to systematically identify, organize and provide insight into patterns of meaning (themes) in the qualitative data. This method involved generating initial codes from the data that is summarizing the participants' statements into concise, meaningful units. These codes were then used to identify and develop themes, review and refine them and finally derive key findings.

*Evaluation of the features.* We used the questions listed in Table 2 to analyze the results of each functionality of the prototype. For this purpose, the statements of all physicians were summarized into meaningful units. As the same questions were asked for each feature of the dashboard, the features were defined as generic terms (see Table 2). During the evaluation, it was observed that participants either supported or opposed to the use of this feature. Consequently, a first subcategory was established, comprising positive and negative responses. Furthermore, a second subcategory was formed for a more comprehensive overview of the data, which included reasons for their support or opposition. Subsequently, the key findings were derived for each feature of the dashboard.

*Evaluation of the visualization.* We used the questions listed in Table 3 to analyze the results of the visualization. For this purpose, the statements of all physicians were summarized into meaningful units. With regard to the topic of prototype exploration (see Table 3), the following subcategories were formed in order to provide a more comprehensive overview of the data: *first impression, interpretation difficulties, preferences between elements, and missing elements*. Subsequently, the key points were extracted. As the participants‘ statements relate to the individual features of the dashboard prototype, it was determined that presenting the results based on the aforementioned subcategories would not be an optimal approach. Instead, the results were presented based on the individual features of the dashboard prototype.

## Results

### Evaluation of the features

Results are presented for each feature of the dashboard prototype. For the following results see Fig. 1, features 2–3 of the dashboard.

In the clinical context, the *injured figure *(2) can be useful for procedural planning, such as organizing necessary interventions and materials, including therapeutic equipment or imaging. However, physicians were concerned about a potential biased approach. They noted that pre-arrival information could unintentionally cause physicians in the ETR to focus on certain areas of the patient's body, potentially resulting in the treatment of other areas that may also require attention. For the physicians surveyed, the *ECG* (3) is primarily useful in the context of circulatory disorders. It indicates the potential presence of a shock and provides information about cardiologic pre-medications and pre-existing therapies. However, some surgeons mentioned, that this information is more relevant for anesthesiologists.

For the following results see Fig. 1, features 4-7 of the dashboard. The physicians also stated that *patient data* (4) such as arrival time, patient name, and date of birth were essential. This information is necessary for scheduling and preparing for upcoming procedures or alerting relevant staff. Additionally, the arrival time enables alerted physicians to manage their time efficiently, attending to other patients during the waiting period before the new patient's arrival. The *ABCDE scheme* (5) allows for more effective preparation of personnel and materials, as well as planning the sequence of procedures in the hospital. For example, it can help the ETR team check the availability of blood reserves in the case of known volume loss, or avoid preparing unnecessary materials, thereby optimizing the use of resources. *Vital signs* (6) are important indicators of a patient’s current health condition and play a crucial role in guiding subsequent medical interventions. The physicians indicated for instance that low oxygen saturation could indicate the possibility of a pneumothorax, leading the ETR staff to anticipate and prepare for a thoracic drainage procedure. The *emergency history* (7) serves a beneficial feature by providing information about the accident, as well as information about the patient's allergies, pre-existing conditions, and medications. These details are crucial for subsequent and follow-up treatments, such as preparing for medication administration or alerting additional specialists.

For the following results see Fig. 1, features 4–7 of the dashboard. In the *multimedia section *(8) of the dashboard, the physicians had mixed opinions about the possibility of a video call feature to facilitate communication between the ambulance and the hospital. On the positive side, this feature would allow direct questioning of the EMS, while also allowing hospital staff to assist the EMS if needed. However, there were concerns about the practicality of this implementation. Emergency physicians at the accident scene are often occupied and may not have sufficient time to engage in video calls. Additionally, the surveyed physicians expressed a desire to visually assess the patient via camera. This would complement the other data presented on the dashboard, providing insights into the patient's current condition, such as burns, motor functions, or signs of epileptic seizures. *Images from the accident scene* are valuable in helping physicians to assess the mechanism of the accident and the severity of injuries. Often, ETR staff receives such images, but they are usually transmitted through other channels, like cell phone messages. Furthermore, physicians stated that they would like to see photos of injuries before a dressing is applied to earlier assess whether additional specialties are required. Secondly, the dressing does not have to be removed for assessment in the ETR and the patient can be taken directly to the operating room.

*Sonography images* can be useful for physicians in preparing staff and materials. For example, the operating room can be prepared in advance, saving time and increasing the efficiency of patient care. However, sonography is primarily relevant for specific cases, such as pneumothorax or assessing the presence of free fluid. It is important to note that the effectiveness of sonography is highly dependent on the examiner, which in turn affects the quality of the images. The surveyed physicians found it helpful to alert selected colleagues using the *alert feature* on the dashboard. Bilateral feedback would be desirable to ensure that the alerted staff acknowledge the alert. Physicians would benefit from receiving audio-visual *notifications* through a notification feature when values on the dashboard change, particularly in life-threatening situations where alarm limits are exceeded and arrival time updates are necessary. Displaying all past changes to patient information in a small window on the dashboard may be useful for documentation purposes, but it does not add any value to the direct care of the patient. During the evaluation, it was observed that physicians at different trauma centers had different perspectives on the usefulness of the information displayed on the dashboard. These perspectives were influenced by factors such as the size and location of the trauma center. Physicians at smaller trauma centers (LTC) appreciated the detailed information provided in the ABCDE scheme and the multimedia section. They considered that this would give them a better understanding of the incoming patient's condition and enable them to make informed decisions about transferring the patient to another hospital with more extensive equipment and treatment options. Some physicians from trauma centers located in more urban areas with shorter transport distance from the accident site to the hospital (STC, RTC) stated that information such as emergency events, the multimedia section, and the notification feature may be helpful, but they also believe that it is more relevant for longer transport distance. For shorter trips in cities, there is limited time to read all the information.

### Evaluation of the visualization

The initial responses of the physicians to the dashboard were mixed. Some found it logically organized, while others felt overwhelmed by the amount of information presented at first glance. However, there was a consensus that with time and familiarity with its features, the dashboard would be effective to work with. During the exploration of the dashboard, the surveyed physicians highlighted the following aspects: Considering the dashboard prototype, physicians identified difficulties in the *ABCDE scheme*, primarily due to the integration of both medical issues and therapies, such as critical bleeding and oxygen therapy. This mix, together with the use of red markings, created confusion. It was unclear whether red markings indicated completion of a therapy or highlighted a medical issue. The physicians expressed a desire for a more distinct organization of medical issues and therapies, as well as an improved wording for the scheme, to enhance clarity and usability. For the *vital signs*, the physicians would like to combine the vital signs as numbers and curves, which is how they are displayed on the monitors in the hospital. To improve clarity, *emergency history* should be presented in a more effective form. If narrative text is used, its length should be minimized. The physicians recommended that *accident images* and the *video call* option should be displayed only when needed. For the *sonography transmission* display, they suggested it would be helpful to indicate the specific body region from which the image was captured. Additionally, they observed that the *injured figure* used in the dashboard did not adequately represent the abdominal region and the spine, indicating a need for a more accurate and comprehensive depiction. With the ability to customize an *individual view* of the dashboard depending on the team or trauma leader, standardization with a common view for all is preferable. Some physicians suggested a customized view of the dashboard on their own smartphone. Physicians are divided on the issue of viewing a *mobile version* of the dashboard on a mobile device (e.g., tablet, smartphone). They see the advantage of mobility within the hospital, being able to access the dashboard from anywhere, for example, to check the arrival time and complete other tasks before the patient arrives. Furthermore, this could facilitate information access for the call service even before they leave home. On the other hand, some physicians consider the mobile version to be redundant, as they prefer to use the dashboard only in the ETR due to the short distances within the hospital.

### Changes to the prototype—iterative process

Based on the findings and feedback from the interviews, an enhanced prototype was developed towards the end of the project, in order to refine the functionality and user experience. Some changes in the new prototype are outlined below, building on the reasons presented in Sects. “Evaluation of the Features” and “Evaluation of the Visualization” (Fig. 2).

The configurability in the *multimedia section* (4) was kept, where different tabs on the right-side display different types of information. As an additional feature, the *alarm feature* (5) for ETR staff was introduced. Alerts can be set based on specific cases, specialties, or individual staff members. The selected staff will be informed via app on mobile devices. This feature was provided to us and integrated into our system (Alarm Dispatcher, Dresden, Germany). Furthermore, a feature of a *patient history window* (3) was also introduced in this prototype to display past changes in the patient's health status and new therapeutic steps. The *ABCDE scheme* (2) was restructured to distinguish between medical issues (red) and therapy (blue), which only appear if they are urgent or currently in progress. For example, if there is no problem under 'A', that column remains empty. In addition, we added a medication administration tracking feature (1) to the patient information section. This allows ETR staff to track what medications have been administered and in what quantities.

## Discussion

The aim of this study was to explore the potential benefits of a new value-based communication interface. Through interviews, physicians evaluated our version of a dashboard that provides real-time patient data transmitted from the ambulance to the hospital. During evaluations of features, physicians concentrate on emergency-relevant information such as the ABCDE protocol, emergency history, and vital signs, as they have a direct impact on patient care. This data provides measurable and precise metrics that are crucial. Some details may be more relevant to anesthesiologists than to surgeons and vice versa. Not all features of the multimedia section are consistently required, such as ultrasound or video call. Therefore, it is advantageous to maintain flexibility in the multimedia section and display only relevant information as needed. When implementing video cameras in ambulances or sending accident images, it is important to carefully plan these functionalities. This ensures an improvement in patient care and compliance with legal and ethical standards. The implementation of the *alert feature* has received positive feedback. In many hospital settings, staff must be individually notified about incoming patients, which can be a time-consuming and labor-intensive process [5, 14]. The bilateral feedback in the alert feature would allow for prompt confirmation of the alerted physicians’ availability or engagement in another emergency, ensuring efficient and effective response in critical situations. The physicians considered a *notification feature* to be useful. It is recognized that an excessive number of alarms from different medical devices can lead to alarm fatigue, making it difficult to distinguish between individual alarms. In the described scenario, where the patient has not yet reached the ETR and no devices can trigger alarms, the dashboard is the primary focus. However, it is essential to ensure that notifications are easily identifiable and do not cause confusion [16, 17].

Considering the results from the evaluation of the visualization, the design and the functionalities necessary for an effective support system can be conceptualized. Initially, the dashboard prototype seemed to overwhelm some physicians due to the abundance of new information. However, its division into three columns can provide a clear overview. The dashboard presents a significant amount of information that needs to be processed initially. It is expected that with frequent use of the dashboard, users will become familiar with it. Although the *individual view* is rather rejected, an advantage is seen in the *mobile version*. In this way, information relevant to each professional could be displayed, while information relevant to all, such as arrival time, could still be maintained. Nevertheless, some physicians reported limited use of mobile devices in certain hospitals. This suggests a potential hesitation to adopt a mobile perspective. However, existing studies underline the benefits of mobile devices in trauma settings for enhancing team communication and patient involvement [18]. The information on the dashboard could be tailored to the specific needs of a hospital, considering size and location of the trauma center. This customization enables hospitals to assess the suitability of their facility for incoming patients. The primary reasons for transferring patients between facilities are medical specialty, higher level of trauma care, and capacity of intensive care unit or operating room [19]. Providing LTCs or RTCs with comprehensive data ahead of a patient's arrival, unnecessary transfers can be minimized, patient care can be optimized and hospital resources can be conserved.

Taking into account the feedback from physicians and the evaluation of our prototype, we propose improvements for a support system. The dashboard’s content could be made configurable, allowing for customization not only in terms of the specific information displayed but also the sequence in which these details are presented. However, it is crucial to maintain a consistent layout once configured, regardless of the clinical setting. Consistency ensures that ETR staff can become familiar with the system's interface. This helps to make navigation intuitive, which improves staff's ability to respond efficiently and effectively in high-pressure situations. It is recommended to include a *mobile version* feature that allows physicians to access critical patient information via mobile devices before arrival. This feature should be tailored to their specialty and informational needs. Moreover, the *notification feature* to inform staff to important updates on the dashboard, designed to be easily identifiable and minimize confusion. The *alert feature* with bilateral feedback is proposed to enhance clinical workflows, ensuring efficient team communication. However, with all of the features that are implemented or being considered for the prototype, it is crucial to keep in mind that the additional workload on EMS to provide information to the ETR should not be added, as time is limited during an emergency response. The scope of the study was primarily physician-centered and lacked important nursing perspectives. Selection criteria for ETR team physicians required refinement for equitable representation across hospitals and specialties to mitigate bias. Unfortunately, due to work schedules or necessary surgeries, some planned participants could not be interviewed or had to be replaced. It can be assumed that due to the data analysis method, this factor had no noticeable effect on the results. Furthermore, a more diverse analysis, including specialty perspectives such as radiology or neurology, would have been particularly useful for individual functionality. Since our interview study focused on the use of the dashboard in a traumatology emergency room, it is important to consider the non-traumatology emergency room as well. Previous studies have demonstrated that meaningful pre-notification can provide effective follow-up care for patients, particularly in the case of stroke and acute myocardial infarction [10, 20]. It is possible that our visualization system needs to be modified to display the most important information about stroke and heart attack. This does not negate the fact that it provides a fundamental enhancement in the treatment process.

The objective of this study was to ascertain the efficacy of transferring data from the ambulance to the ETR in advance of the patient’s arrival, thereby affording the ETR staff the opportunity to prepare for the imminent arrival of their patient. However, a comprehensive view of the data transfer process suggests that future enhancements could also include the integration of patient and medical data into the hospital's patient management system. From the perspective of hospital staff, this approach can alleviate the burden associated with regulatory processes, as it enables the automated documentation of patient usage and registration within the hospital. Additionally, it offers protection against potential media disruptions [21, 22]**.** Adequate transfer of pre-hospital information to hospitals and storing the data in the patient data management system is critical for both health systems and policy frameworks. From a healthcare perspective, it improves the quality and safety of care, while politically coordinated measures and legal frameworks should support its implementation. Hence, digitalization of emergency medical services is a key step in meeting the challenges of modern emergency medicine and ensuring a connected healthcare system [23–25]. In the context of our study and the project behind it, our visualization system may add value to prehospital communication because it integrates different data and provides added value within the clinic, e.g., alerting clinicians. Existing systems such as the NIDApad are also integrated. In addition to our application, the consistent and comprehensive data collection can enable many other applications in the future, such as automated documentation, AI-based support, and more [26]. This study aligns with existing literature highlighting the critical role of technology in overcoming prehospital-to-hospital communication gaps, and underscores a general movement in healthcare industry toward using digital solutions for more efficient emergency care and patient management. Through the development and evaluation of a dashboard prototype, this research contributes to the dialogue on the use of digital technology, and supports the continued development of these technologies to effectively meet clinical needs.

## Conclusion

In an ETR setting, physicians must gather information and act simultaneously, if possible. Acting is beneficial for the patient. If information is available in advance, physicians are able to act directly and with less disruption. A real-time data transfer from the ambulance to ETR, coupled with the dashboard visualization, has the potential to assist physicians in their daily tasks, thereby improving the quality of patient care A configurable version of the dashboard tailored to the specific needs of each hospital or specialist (as mobile version), allows for transfer of only essential information. The results of our investigation underscore the importance of customizable dashboards. They should meet the diverse needs of emergency teams and provide rapid access to critical patient information to make patient care processes more efficient. The practical applicability and support in daily clinical practice requires further investigation. The next step should be a re-evaluation of the second prototype by professionals and another prototype implementation phase. A field trial of the dashboard prototype could effectively determine its practicality and relevance in real-world clinical scenarios.

## References

[1] Ruchholtz S, Mand C, Lewan U, Debus F, Dankowski C, Kühne C, Siebert H (2012) Regionalisation of trauma care in Germany: the ‘TraumaNetwork DGU®-Project.’ Eur J Trauma Emerg Surg 38(1):11–17. 10.1007/s00068-011-0166-6 26815667 10.1007/s00068-011-0166-6

[2] Sieber F, Kotulla R, Urban B, Groß S, Prückner S (2020) Entwicklung der Frequenz und des Spektrums von Rettungsdiensteinsätzen in Deutschland. Notf Rettungsmedizin 23(7):490–496. 10.1007/s10049-020-00752-1

[3] Frink M, Kühne C, Debus F, Pries A, Ruchholtz S (2013) Das Projekt TraumaNetzwerk DGU®: Zielsetzung, Konzeption und bisher Erreichtes. Unfallchirurg 116(1):61–73. 10.1007/s00113-012-2326-5 23307433 10.1007/s00113-012-2326-5

[4] Deutsche Gesellschaft für Unfallchirurgie, Ed (2019) Weißbuch Schwerverletztenversorgung, 3rd edn. Berlin

[5] James MK, Clarke LA, Simpson RM, Noto AJ, Sclair JR, Doughlin GK, Lee S-W (2019) Accuracy of pre-hospital trauma notification calls. Am J Emerg Med 37(4):620–626. 10.1016/j.ajem.2018.06.058 30041910 10.1016/j.ajem.2018.06.058

[6] Handolin LE, Jääskeläinen J (2008) Pre-notification of arriving trauma patient at trauma centre: a retrospective analysis of the information in 700 consecutive cases. Scand J Trauma Resusc Emerg Med 16(1):15. 10.1186/1757-7241-16-15 19019252 10.1186/1757-7241-16-15 PMC2612016

[7] Eder PA, Dormann H, Krämer RM, Lödel SK, Shammas L, Rashid A (2019) Telemedizinische Voranmeldung durch den Rettungsdienst bei Schwerverletzten: Fallbericht eines Verkehrsunfalls. Notf Rettungsmedizin 22(1):37–44. 10.1007/s10049-018-0436-5

[8] Wurmb T, Jansen H, Böttcher M, Kredel M, Wunder C, Gehrmann A, Roewer N, Muellenbach R (2014) Schockraumaufnahme schwerverletzter oder kritisch kranker Patienten: Geschätzte und tatsächliche Eintreffzeiten im Vergleich. Unfallchirurg 117(3):242–248. 10.1007/s00113-013-2529-4 24408199 10.1007/s00113-013-2529-4

[9] Schlingloff F, Marian T, Seeger I, Steffen T (2023) Pilotprojekt „Telenotfallmedizin Niedersachsen“: Telenotfallmedizin in einer ländlichen Gegend im deutschen Mittelgebirge. Notf Rettungsmedizin 26(5):356–362. 10.1007/s10049-022-01086-w

[10] Eder PA, Laux G, Rashid A, Kniess T, Haeusler KG, Shammas L, Griewing B, Hofmann S, Stangl S, Wiedmann S, Rücker V, Heuschmann PU, Soda H, Stroke Angel Study Group (2021) Stroke angel: effect of telemedical prenotification on in-hospital delays and systemic thrombolysis in acute stroke patients. Cerebrovasc Dis 50(4):420–428. 10.1159/000514563 10.1159/000514563 33774614

[11] Ortivus MobiMed AB (2024) A product suite designed to connect the prehospital care chain. Ortivus. https://www.ortivus.com/ortivus/mobimed-unity/. Accessed Jun 30 2024

[12] Pabst T, Stegemann D, Georgi C, Kasparick M, Suleder J, Neumuth T, Rockstroh M (2021) TeleSDC: concept for ISO/IEEE 11073 SDC in telemedicine across unreliable networks. Curr Dir Biomed Eng 7(2):403–406. 10.1515/cdbme-2021-2102

[13] Rockstroh M, Suleder J, Bockelmann C, Gaebel J, Georgi C, Neumuth T, Will A, Wendlandt R (2020) Towards an integrated emergency medical care using 5G networks. Curr Dir Biomed Eng 6(3):5–8. 10.1515/cdbme-2020-3002

[14] Schatz A, Osterhoff G, Joeres F, Neumuth T, Rockstroh M (2023) Data exchange between ambulance and trauma center: interview study about the needs of emergency trauma room staff. In: Pfeifer B, Schreier G, Baumgartner M, Hayn D (eds) Studies in health technology and informatics, IOS Press. 10.3233/SHTI230022 10.3233/SHTI230022 37172163

[15] Deutsche Gesellschaft für Unfallchirurgie e.V (2022) S3-leitlinien polytrauma/schwerverletzten-behandlung (AWMF registernummer 187-023, Version 4.1, Dezember 2022)”

[16] Roche TR, Braun J, Ganter MT, Meybohm P, Herrmann J, Zacharowski K, Raimann FJ, Piekarski F, Spahn DR, Nöthiger CB, Tscholl DW, Said S (2021) Voice alerting as a medical alarm modality for next-generation patient monitoring: a randomised international multicentre trial. Br J Anaesth 127(5):769–777. 10.1016/j.bja.2021.07.015 34454710 10.1016/j.bja.2021.07.015

[17] Joseph B, Pandit V, Khreiss M, Aziz H, Kulvatunyou N, Tang A, Wynne J, O’Keeffe T, Friese RS, Weinstein RS, Rhee P (2013) Improving communication in level 1 trauma centers: replacing pagers with smartphones. Telemed E-Health 19(3):150–154. 10.1089/tmj.2012.0114 10.1089/tmj.2012.0114 23384333

[18] Lindeque BG, Furness ND, Bradford OJ, Paterson M (2013) Tablets in trauma: using mobile computing platforms to improve patient understanding and experience. Orthopedics 36(3):205–208 23464939 10.3928/01477447-20130222-06

[19] Spering C, Bieler D, Ruchholtz S, Bouillon B, Hartensuer R, Lehmann W, Lefering R, Düsing H, and for Committee on Emergency Medicine, Intensive Care and Trauma Management (Sektion NIS) of the German Trauma Society (DGU) (2023) Evaluation of the interhospital patient transfer after implementation of a regionalized trauma care system (TraumaNetzwerk DGU®) in Germany. Front Med 10:1298562. 10.3389/fmed.2023.1298562 10.3389/fmed.2023.1298562 PMC10684689 38034545

[20] Marcolino MS, Maia LM, Oliveira JAQ, Melo LDR, Pereira BLD, Andrade-Junior DF, Boersma E, Ribeiro AL (2019) Impact of telemedicine interventions on mortality in patients with acute myocardial infarction: a systematic review and meta-analysis. Heart 105(19):1479–1486. 10.1136/heartjnl-2018-314539 31253696 10.1136/heartjnl-2018-314539

[21] Fischer M, Kehrberger E, Marung H, Moecke H, Prückner S, Trentzsch H, Urban B (2016) Eckpunktepapier 2016 zur notfallmedizinischen Versorgung der Bevölkerung in der Prähospitalphase und in der Klinik. Notf Rettungsmedizin 19(5):387–395. 10.1007/s10049-016-0187-0

[22] Turer RW, Smith GC, Mehkri Do F, Chou A, Fowler R, Idris AH, Lehmann CU, McDonald SA (2022) Improving emergency medical services information exchange: methods for automating entity resolution. In: Deserno TM, Haghi M, Al-Shorbaji N (eds) Studies in health technology and informatics. IOS Press. 10.3233/SHTI220004. 10.3233/SHTI220004 35593755

[23] Neumuth T, Möllenhoff C, Rockstroh DM (2024) Whitepaper: ‘Telemedizin im Rettungsdienst: Weichenstellung für eine digitale Zukunft’

[24] Eder PA et al (2023) Digitales Notfallmanagement im Netzwerk der Akut- und Notfallversorgung. Eckpunktepapier des Expertenrats des ZTM. Notf Rettungsmedizin. 10.1007/s10049-023-01241-x

[25] Krafft TA, Neuerer M, Böbel S, Reuter-Oppermann M (2022) Notfallversorgung and Rettungsdienst in Deutschland: Partikularismus vs Systemdenken. Bertelsmann Stiftung, Gütersloh-Winnenden

[26] Eder PA, Rashid A (2023) Telemedicine for prehospital trauma care: a promising approach. In: Aseni P, Grande AM, Leppäniemi A, Chiara O (eds) The high-risk surgical patient. Springer International Publishing, Cham, pp 683–689. 10.1007/978-3-031-17273-1_61

